# Evidence of auditory insensitivity to vocalization frequencies in two frogs

**DOI:** 10.1038/s41598-017-12145-5

**Published:** 2017-09-21

**Authors:** Sandra Goutte, Matthew J. Mason, Jakob Christensen-Dalsgaard, Fernando Montealegre-Z, Benedict D. Chivers, Fabio A. Sarria-S, Marta M. Antoniazzi, Carlos Jared, Luciana Almeida Sato, Luís Felipe Toledo

**Affiliations:** 1 Laboratório de História Natural de Anfíbios Brasileiros (LaHNAB), Departamento de Biologia Animal, Instituto de Biologia, Universidade Estadual de Campinas, Campinas, São Paulo, 13083-862 Brazil; 2 Department of Physiology, Development & Neuroscience, University of Cambridge, Downing Street, Cambridge, CB2 3EG United Kingdom; 3 Department of Biology, University of Southern Denmark, Campusvej 55, DK-5230 Odense M, Denmark; 4 Bioacoustics and Sensory Biology Lab, School of Life Sciences, Joseph Banks Laboratories, University of Lincoln, Green Lane, Lincoln, LN6 7DL United Kingdom; 5 Laboratory of Cell Biology, Instituto Butantan, São Paulo, 05503-900 Brazil

## Abstract

The emergence and maintenance of animal communication systems requires the co-evolution of signal and receiver. Frogs and toads rely heavily on acoustic communication for coordinating reproduction and typically have ears tuned to the dominant frequency of their vocalizations, allowing discrimination from background noise and heterospecific calls. However, we present here evidence that two anurans, *Brachycephalus ephippium* and *B. pitanga*, are insensitive to the sound of their own calls. Both species produce advertisement calls outside their hearing sensitivity range and their inner ears are partly undeveloped, which accounts for their lack of high-frequency sensitivity. If unheard by the intended receivers, calls are not beneficial to the emitter and should be selected against because of the costs associated with signal production. We suggest that protection against predators conferred by their high toxicity might help to explain why calling has not yet disappeared, and that visual communication may have replaced auditory in these colourful, diurnal frogs.

## Introduction

Communication is defined as the transmission of a signal from a sender to a receiver such that the sender benefits from the response of the receiver. For a signal to elicit a change in the receiver’s behaviour, the receiver must be able to detect the signal and decipher its information content. In many animals, communication is used in a sexual context to convey information about species, motivational state, mate-quality and location of the signaller. Sensory receptors co-evolve with signals for efficient detection in any particular environment^1,2^.

**Figure 1:**
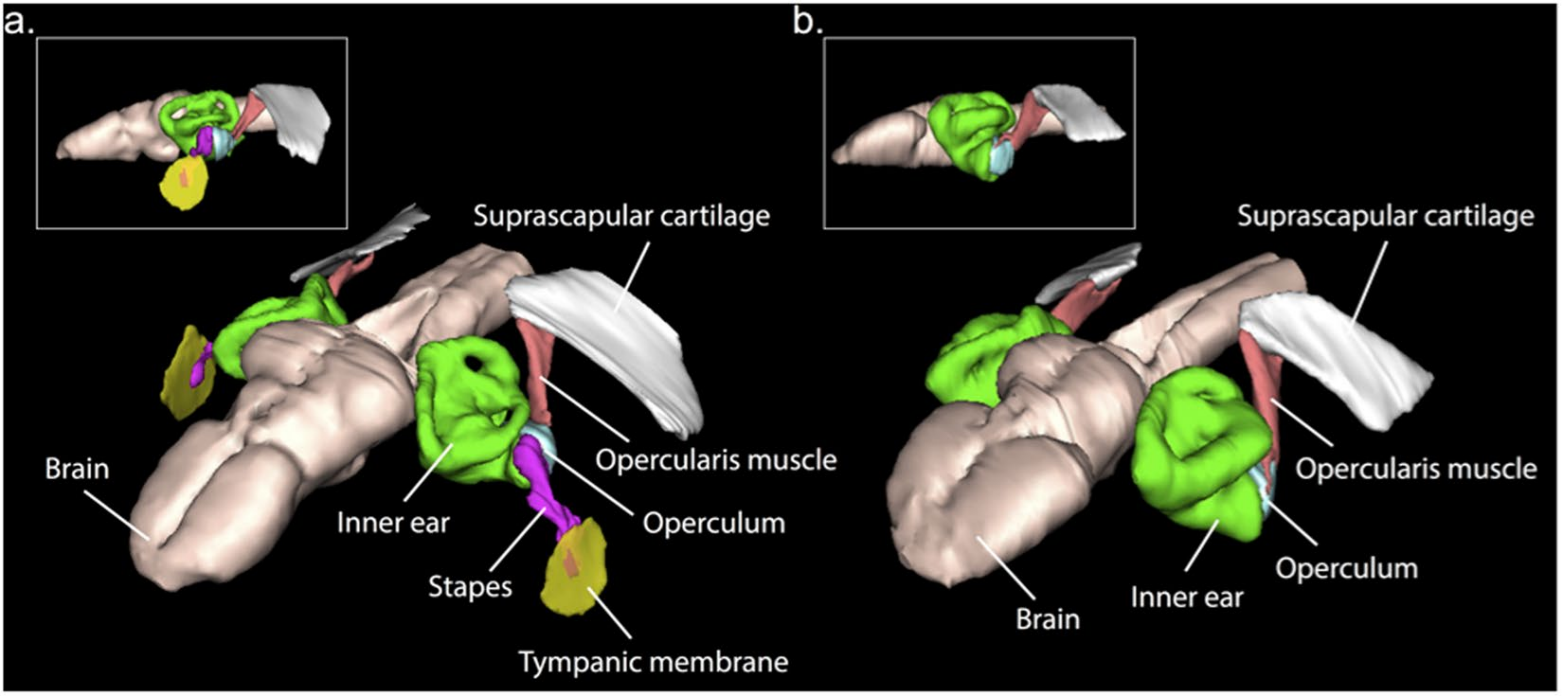
Middle ear structures in “eared” and “earless” frogs. Three-dimensional reconstructions (anterolateral view), made from micro-computed tomography data, of the middle ear structures and brains of (**a**) the “eared” frog *Ischnocnema parva*, and (**b**) the “earless” pumpkin toadlet *Brachycephalus ephippium*. Lateral views are presented in upper panels.

Most anurans (frogs and toads) rely heavily on acoustic communication for reproduction and their hearing structures are well adapted to detect conspecific calls on land. Their middle ear, which in its most complete form includes a tympanic membrane, air-filled middle ear cavity, extrastapes and stapes^3^, mechanically amplifies and transmits airborne sound to the inner ear. This transmission pathway is particularly important at sound frequencies above one kilohertz (kHz), where the impedance mismatch (and thus the energy loss during transmission of vibrations) between air and body tissues is high^4,5^. Additionally, their inner ear sensitivity range typically matches the dominant frequency of their vocalizations^6^, allowing them to discriminate conspecific calls from background noise and heterospecific calls occurring at different frequencies. Within the anuran inner ear, two sensory organs are largely responsible for the perception of airborne sounds: the amphibian papilla (AP), sensitive to low and mid-frequencies (typically 50 Hz to 1 kHz), and the basilar papilla (BP), sensitive to higher frequencies (above 1 kHz)^7^. The dominant frequencies of anuran calls may fall into either of these two organs’ sensitivity ranges. Some anurans, inappropriately termed “earless”^8,9^, lack tympanic middle ears, but inner ears are retained (Fig. 1). “Earless” frogs may channel sound to their inner ears through extra-tympanic pathways involving the lungs, mouth cavity or cranial bones^10,11^, and all “earless” species studied so far are able to hear their own vocalizations^12^.

**Figure 2:**
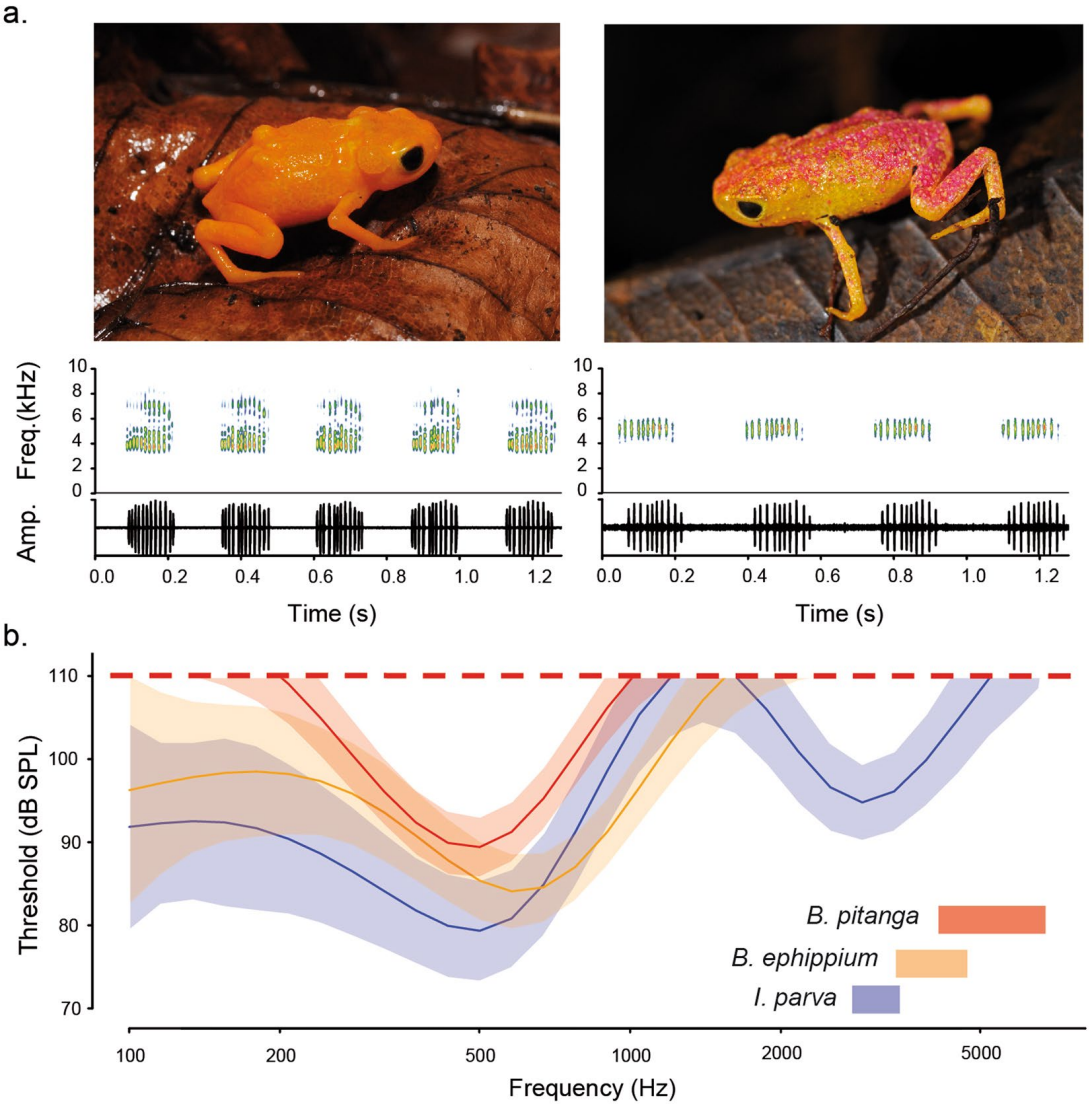
Vocalizations and hearing thresholds in pumpkin toadlets. (**a**) Vocalizations of *Brachycephalus ephippium* (left) and *B. pitanga* (right) are represented by spectrograms (upper panels; kHz; high, intermediate and low sound intensities are represented in red, green and blue, respectively) and oscillograms (lower panels; relative amplitudes), (**b**) Hearing sensitivity threshold curves for *B. pitanga* (red, n = 4), *B. ephippium* (orange, n = 6), and *Ischnocnema parva* (blue, n = 3). Solid lines indicate species averages with 95% confidence intervals shaded. Dashed red line represents maximum sound pressure level employed during experiments (110 dB). Colour-coded rectangles show frequency ranges of species’ vocalizations (4.26–6.98 kHz for *B. pitanga*, 3.38–4.84 kHz for *B. ephippium* and 2.87–3.53 kHz for *I. parva*).

Pumpkin toadlets (*Brachycephalus* spp.) are a radiation of tiny (7–15 mm snout-vent length) and often brightly coloured “earless” anurans that live in leaf litter in Brazil’s Atlantic forest^8,9,13^. During the breeding season, individuals of *Brachycephalus ephippium* and *B. pitanga* (Fig. 2a) are found in restricted forest patches where males call from low perches or the leaf litter^14^ (Video S1). The high-frequency, low-amplitude calls of pumpkin toadlets should undergo significant energy loss when transmitted from ambient air to body tissues and require a sensitive hearing apparatus to be detected. The lack of a tympanic middle ear in these frogs thus raises the question of how they can detect such sounds. In this study, we integrated field call recordings and playback, auditory brainstem response (ABR) and laser Doppler vibrometry (LDV) experiments to test whether *Brachycephalus ephippium* and *B. pitanga* can hear their own vocalizations. We then explored their inner ear anatomy using histological serial sectioning and three-dimensional model reconstruction.

## Results

Pumpkin toadlets’ vocalizations have relatively high dominant frequencies for anurans, 3.94 ± 0.24 kHz for *B. ephippium* (n = 5 males) and 5.43 ± 0.30 kHz for *B. pitanga* (n = 8 males; Fig. 2a). These calls are remarkably quiet for anurans^14^ (Video S1), even when considering their minute size: 47.0 ± 5.7 dB SPL and 57.6 ± 1.8 dB SPL at 50 cm distance for *B*. *ephippium* (n = 3) and *B. pitanga* (n = 8), respectively. By comparison, male *Cacosternum boettgeri* (18–19 mm SVL) produce calls up to 108 dB SPL at a distance of 50 cm^15^ from shallow water, and male *Allobates femoralis* (25.8 mm SVL^16^) call from the ground or vegetation (similarly to male *Brachycephalus* spp.) at an amplitude up to 92 dB at 50 cm^17^.

Playback of specific advertisement calls to male *B. pitanga* in the field did not yield any change in calling behaviour or posture (n = 8; Figure S1). Phonotaxis experiments on gravid female *B. pitanga* were also negative (n = 7; Figure S2), although we cannot be certain that these animals were fully receptive to males at the time of the experiment. Although these (negative) results must be treated with caution (see sup. mat.) because of the low sample size and the uncertain receptivity of the females, they suggest that either that the toadlets heard the calls but did not respond actively, or that they could not hear the calls.

We tested these hypotheses by measuring hearing sensitivity through the auditory brainstem response (ABR)^18^ method in both sexes of the two pumpkin toadlet species (four male and two female *B. ephippium* and three male and one female *B. pitanga*). These animals lack tympanic membrane, extrastapes, stapes and middle ear cavity and hence are considered “earless”, although they retain an otic operculum and inner ear (Figure 1)^8^. Three males of a similarly sized, “eared” species of the sister genus, *Ischnocnema parva*, were also examined for comparison. Two sensitivity peaks were shown for *I. parva*, presumably corresponding to the sensitivity ranges of the AP (200–1200 Hz) and the BP (2000–3000 Hz; Fig. 2b). In the *Brachycephalus* species, only low frequencies (200–1200 Hz) yielded a response, with a sensitivity curve similar to the low-frequency sensitivity found in *I. parva* (Fig. 2b). While the *Brachycephalus* species tested are not completely insensitive to airborne sounds, they are insensitive to high frequencies (above 1 kHz), and thus to their own 3.7–5.7 kHz calls (Fig. 2a).

**Figure 3:**
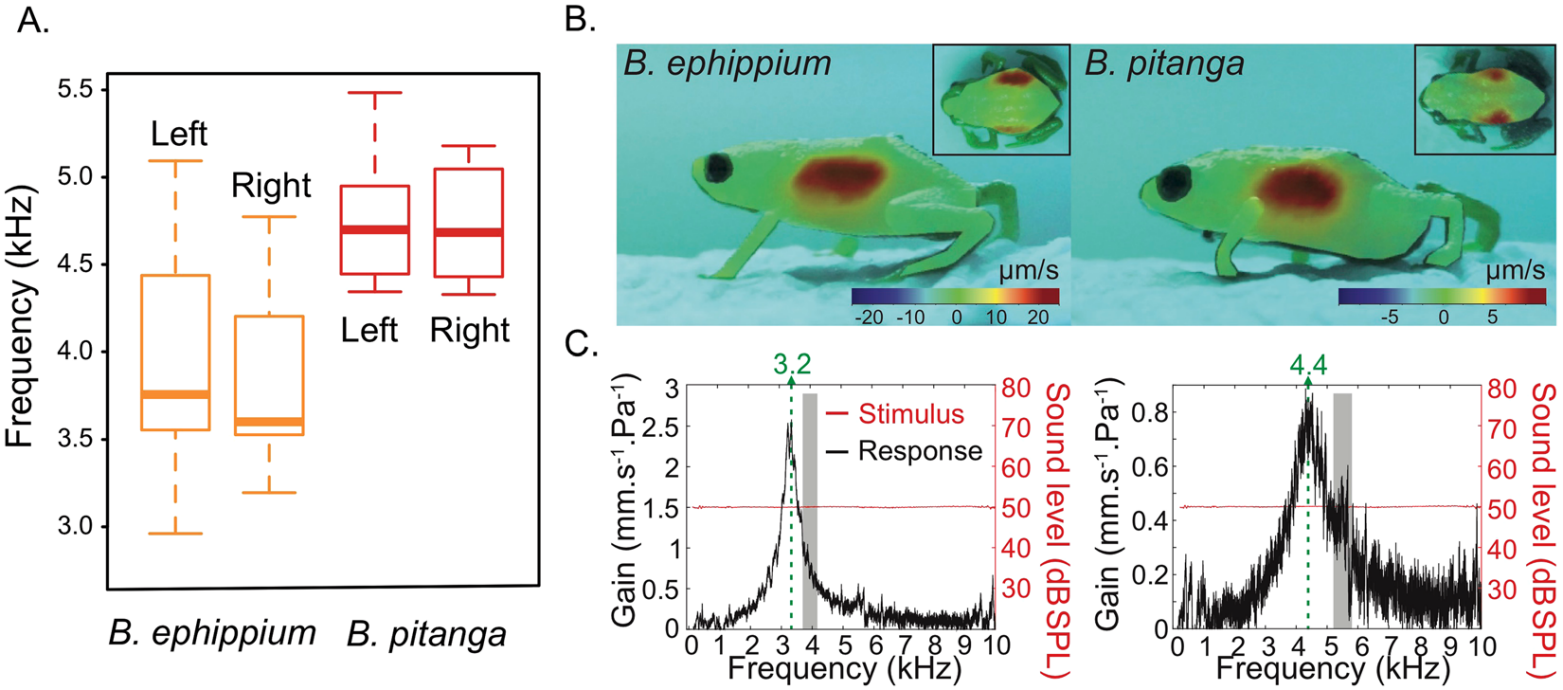
Skin vibrational response to airborne sounds in pumpkin toadlets. (**A**). Resonant frequency of the skin overlying the lungs in *B. ephippium* (n = 10) and *B. pitanga* (n = 11). Means (thick solid lines), quartiles (box edges) and extreme values (whiskers) for left and right sides are given, (**B**). Skin vibrational response to airborne sounds (1.5–20 kHz) for *B. ephippium* (left) and *B. pitanga* (right). Areas of high vibration amplitude are indicated in red (**C**). Skin vibrational response (average of all points measure on the surface) of *B. ephippium* (left) and *B. pitanga* (right), presented as velocity gain (transfer function of the laser signal and stimulus reference) from 150 Hz to 10 kHz. Specific advertisement call frequency ranges (4.26–6.98 kHz for *B. pitanga* and 3.38–4.84 kHz for *B. ephippium*) are shaded in grey.

We used micro-scanning laser Doppler vibrometry to investigate whether acoustic insensitivity in these species is due to poor sound transmission from the air to pumpkin toadlets’ bodies. We measured the vibratory responses of the lateral, dorsal and ventral surfaces of the toadlets’ bodies to airborne sound (from 0.15 to 20 kHz) in ten *B. ephippium* and 11 *B. pitanga*. We also tested nine *I. parva* (Figure S3). No significant vibration was recorded for the skin overlying the otic region in earless species (Fig. 3 B), nor the skin underlying the mouth cavity, a proposed alternate channel of sound transmission in a similarly-sized frog species^11^. However, the body surface overlying the lungs exhibited a clear vibratory response to airborne sound (Fig. 3 B), with a resonant frequency close to that of the specific calls (Fig. 3 C). Sound at vocalization frequencies can therefore, at least partially, pass from the surrounding air into the toadlets’ bodies, although the ABR measurements did not show any sensitivity to these frequencies. The partial frequency matching between the calls and the resonance of the toadlets’ body walls suggests that the lungs and body walls may be involved in emission of the calls.

**Figure 4:**
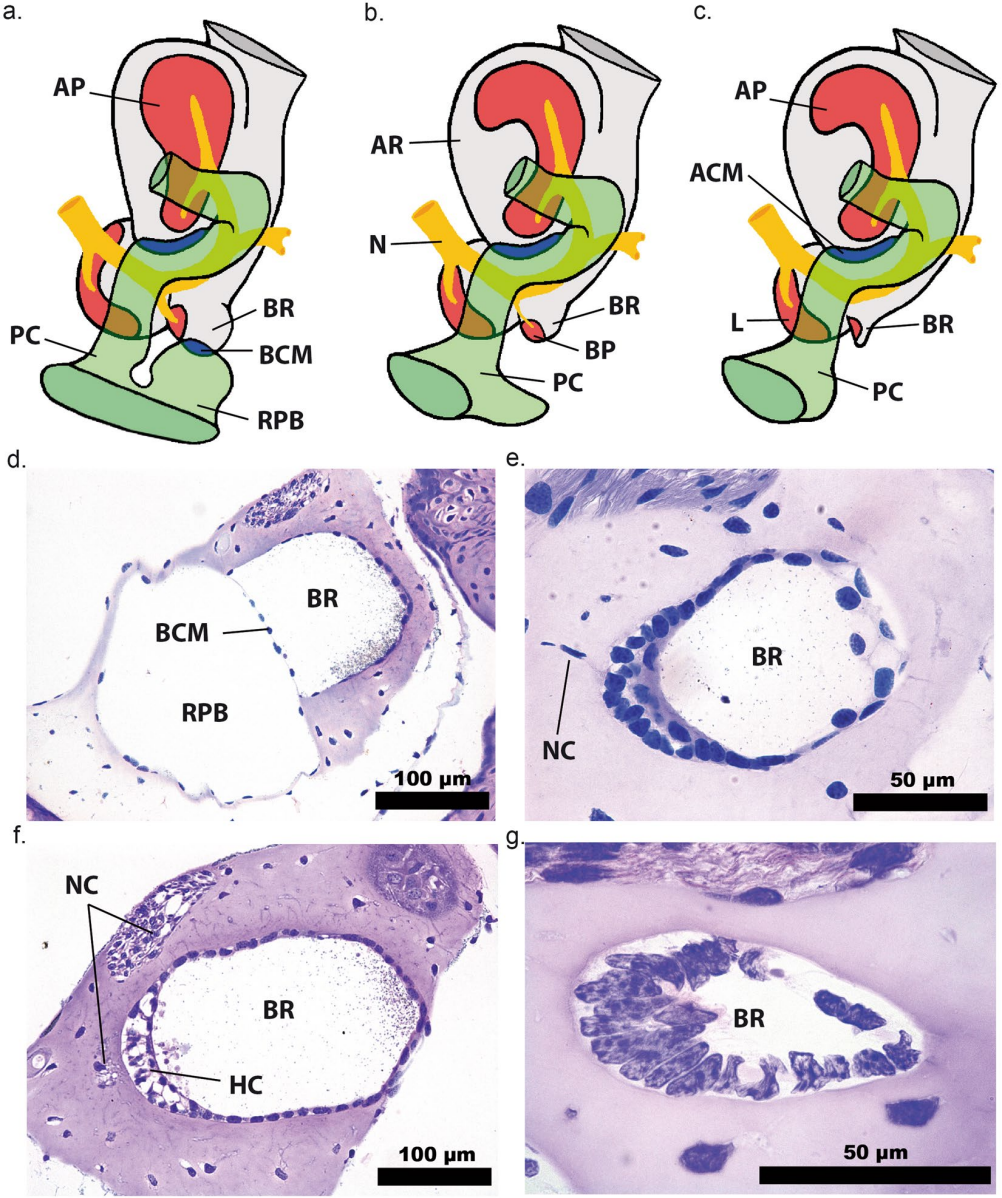
The inner ear in pumpkin toadlets. Diagrammatic representations of right inner ear structures of *Ischnocnema parva* (**a**), *Brachycephalus pitanga* (**b**) and *B. ephippium* (**c**) in approximately posterodorsal views. Note that *I. parva* has a typical anuran basilar papilla, but elements of this are reduced in the two *Brachycephalus* species. Photomicrographs of histological sections of basilar recesses of *I. parva* (**d** and **f**), *B. pitanga* (**e**) and *B. ephippium* (**g**). ACM: amphibian recess contact membrane, AP: amphibian papilla, AR: amphibian recess, BCM: basilar recess contact membrane, separating the basilar recess (filled with endolymph; light grey) from the *recessus partis basilaris* (filled with perilymph; green), BP: basilar papilla, BR: basilar recess, HC: hair cells, L: lagena, N: branch of eighth cranial nerve, NC: nerve cells, PC: periotic canal, RPB: *recessus partis basilaris*.

Histological sections of the inner ears show that the pumpkin toadlets’ basilar papillae are underdeveloped. In frogs, the sensory cells (hair cells) of the basilar papilla lie in the wall of an endolymphatic diverticulum called the basilar recess^19^ (Fig. 4). The end of the basilar recess typically meets a perilymphatic channel called the *recessus partis basilaris* (RPB), the two being separated by a very thin ‘contact membrane’^19^ (Fig. 4a and d). It is thought that the contact membrane and the RPB together provide a low-impedance outlet pathway, the presence of which increases the proportion of sound energy within a particular frequency range which can enter the basilar recess to be detected by the hair cells there^20^. In both *B. ephippium* and *B. pitanga*, the basilar recess is present but there is no contact membrane and no RPB (Fig. 4b and c). Additionally, the hair cells in the basilar recess appear disorganized: they do not present the typical columnar shape and lack well-defined hair bundles (Fig. 4g and f), as opposed to *I. parva* (Fig. 4g and f). Innervation of the basilar papilla is lacking in *B. ephippium* (Fig. 4c and g) and extremely reduced in *B. pitanga* (Fig. 4b and e). No BP tectorial membrane was found in either species, but difficulties in preserving this delicate structure prevent us from concluding for certain that it is missing. In other frogs, these structures are presumed necessary for the perception of frequencies above 1 kHz^7^. Lacking functional basilar papillae, these two *Brachycephalus* species are expected to lack high-frequency auditory perception, regardless of the preceding transmission pathway. This explains the ABR results. Although the loss of a basilar papilla has been documented in certain non-anuran amphibians which do not vocalize (some caudates and caecilians; see review^21^), this is the first time that the degeneration of this sensory organ has been documented in anurans. In summary, both anatomical and ABR data suggest that sounds within the calls’ frequency ranges are not processed by the auditory pathway in these species.

*Brachycephalus hermogenesi*, a brown-coloured species of the same genus, also produces high-frequency calls (Dominant frequency = 7 kHz, male SVL = 8.7 mm)^22,23^. Histological sections show that, contrary to *B. ephippium* and *B. pitanga*, this species possesses a fully developed inner ear (Figure S4). Although we did not test hearing sensitivity in this species, the BP appears functional as it is separated from an RPB by a contact membrane, it clearly possesses a tectorial membrane, contains hair cells of typical appearance, and is innervated by a larger nerve branch (Figure S4). The presence of a complete inner ear within the genus indicates that the reduction of the BP and consequent loss of high-frequency hearing in *B. ephippium* and *B. pitanga* is a relatively recent evolutionary event (less than 29.4 million years ago^24^). The existence of homologous high-frequency calls in *B. hermogenesi* and other *Brachycephalus* species^22,25–27^ places the emergence of high-frequency calls prior to the loss of high-frequency sensitivity in pumpkin toadlets, reinforcing the hypothesis that the low-amplitude calls in *B. ephippium* and *B. pitanga* are vestigial.

## Discussion

The loss of high-frequency auditory sensitivity in pumpkin toadlets strongly suggests that these species are unable to perceive their own calls. As signal production is energetically costly and sound may attract predators and parasites, ineffective signals should be strongly selected against. Calling behaviour may be maintained because the visual components of calling (for example, movement of the vocal sac^28–30^, see Video S1), originally a by-product of call production, became the selected advertisement signal thereby making the acoustic output in turn a signal by-product^31^. This evolutionary scenario is plausible because *B. ephippium* and *B. pitanga* are diurnal, brightly coloured and show mouth-gaping and arm-waving behaviours^14^ (Video S2). These behaviours have been described by Pombal and colleagues as aggressive intra-specific signals^14^, but they remain largely unexplored.

The persistence of their calls may alternatively relate to the fact that pumpkin toadlets are highly toxic, containing tetrodotoxin-like peptides in their skin and internal organs^32^. The risk of predation when calling is consequently reduced and this behaviour, if not strongly selected against, may be retained through evolutionary inertia. Chemical communication has been demonstrated in other earless anurans^33^ and may potentially play a part in pumpkin toadlets’ signalling behaviour, although this remains to be investigated. Finally, the loss of inner ear structures (and thereby reduction in hearing sensitivity range) may be the result of genetic mutations (no longer selected against) and drift, or could be a side-effect of other selected traits (eg. the important hyperossification of the skull which is present in both species) affecting the ears’ development. The unique status of acoustic communication in *Brachycephalus* spp. is likely to provide further insights into the evolution and degeneration of acoustic communication systems in vertebrates.

## Methods

### Animals

Animals were visually and acoustically located and collected at the Parque Estadual da Serra do Mar, state of São Paulo, Brazil between February and April 2016 (IBAMA collecting permit, 27745-9; COTEC state permit: 468/2015 D028/2015). All animals used were classified as adults because they were of adult size, several males were collected while calling, and females had mature oocytes in their ovaries (visible through the skin; some individuals were also dissected after the experiments). Animals were kept in terraria each containing up to ten individuals, at 23 °C with natural light from 06:00 to 18:00. A high humidity level was maintained by misting the terraria every other day. Larger individuals were fed fruit flies (*Drosophila melanogaster*); smaller ones fed on micro-invertebrates such as springtails (*Collembola* spp.) present in the leaf litter, which was changed regularly. Live animals were imported to Europe (export permit: 16BR019765/DF; veterinary entry document: CVEDA.GB.2016.0002739-V1) to conduct vibrometry and auditory brainstem experiments. All experiments were performed in accordance with relevant guidelines and regulations, and experimental protocols were approved by the College of Science Research Ethics Committee for the LDV experiment (Ethics permit CORSEC. 111), and the Danish National Animal Experimentation board (permit N°2015-15-0201-00619) for the auditory brainstem experiment.

### Behaviour of pumpkin toadlets

*Brachycephalus ephippium* and *B. pitanga* are small frog species (1.5 cm and 1 cm snout-vent length, respectively) living in the leaf litter of the Brazilian cloud forest at 800–1500 m above sea level^22,26,27^. They are not dependent on water sources as they lack an aquatic larval stage and froglets hatch directly from eggs laid on the ground (see Video S2). During the rainy season (November - March), adults are found during the day in restricted forest patches. Calling males are found perched on low vegetation (below 10 cm height) or on the leaf litter (Video S1). Male *B. pitanga* observed in their natural habitat called in high density (up to five active individuals per square meter), regularly changed calling post and did not exhibit territorial or lekking behaviour. In two instances, male *B. pitanga* were observed to stop calling when a female approached. The males then started leading the females to a hidden location, stopping from time to time and turning back towards the female. This interaction would last over 30 minutes as these frogs walk particularly slowly. Amplexus happened hidden from the observer. Both species use mouth-gaping and arm-waving behaviours in what seemed to be aggressive or defence responses^14^ (Video S2). These visual signals may be directed at humans, other potential predators, or conspecifics^14^, but this behaviour was only observed in response to human observers during the present study.

### Acoustic recordings and playback experiments

Vocalizations of male *Brachycephalus pitanga* were recorded in the Parque Estadual da Serra do Mar, núcleo Santa Virgínia (S 23°20′11.5, W 45°08′45.1, 729 m asl.), state of São Paulo, Brazil, in October 2014 between 07:00 and 17:00. Recordings were made at 92–156 cm distance from the calling male, with a Sennheiser ME64 microphone and an Olympus LS-100 recorder at a sampling rate of 44.1 kHz. Exact distance between the microphone and the calling male was measured with a Stanley TLM100i laser meter (precision 3 mm). Sound pressure level (SPL) was measured with an Instrutherm DEC-490 SPL meter on A-weighting (precision 1.4 dB) and FAST mode, and we took the maximal SPL value during a call. Maximal SPL of males’ advertisement calls was 51.6 ± 1.8 dB SPL at 1 m distance (n = 8). These calls were often masked by bird songs or insect stridulations (Video S1.), in which case the SPL measurements were discarded.

Ten recordings of five males were selected and used in randomized order for playback experiments conducted in the same population *in situ* in November 2015 between 7:00 and 17:00. Eight calling male *B. pitanga* were audio or video recorded for ten minutes. Randomized calls were then broadcast continuously for an additional ten minutes and changes in behaviour were monitored. Calling activity, measured as the percentage of time spent calling continuously, was compared between control and playback treatments (Figure S1). No behaviour typical of playback response in anurans (such as orientation to sound, phonotaxis or increase in calling activity) was observed during playback. Playback experiments were also conducted with gravid female *B. pitanga* (n = 7). Although collecting females in amplected pairs would have given us a greater confidence in the receptivity of the females to males’ vocalizations, encounters of amplected pairs were too rare to be used for the experiment.

Females were thus selected when large white eggs could be seen through their abdomen. Trials were conducted in the field, close to the zone of *B. pitanga* activity, in a 1 m diameter Styrofoam circular arena between 11:00 and 17:30. The female was placed under an opaque plastic cup in the middle of the arena. Playback was broadcast from one side of the arena (changing the speaker location for each female in a randomized order) and the cup was lifted after 5 min habituation. Females were monitored via a camcorder transmitting images in real-time to the observer, who was hidden from the focal female. Each female was monitored until she reached the side of the arena or she hid under the leaves for 10 min (Figure S2). For all playback experiments, stimuli were calibrated at 50 dB SPL at 1 m (equivalent to 56 dB SPL at 50 cm) before each trial.

SPL of male *B. ephippium* were measured in a similar manner at biological reserve Serra do Japi (S 23°13′50.8, W 46°56′8.6, 1000 m asl.), state of São Paulo, Brazil on November 8th 2016. Maximal SPL of males’ advertisement calls was 41.0 ± 5.7 dB SPL at 1 m distance (n = 3). Recordings are deposited at Fonoteca Neotropical Jacques Vielliard, Campinas, Brazil (FNJV 33193–33203), accessible at: http://www2.ib.unicamp.br/fnjv/.

### Auditory brainstem response tests

Tests were successfully performed in six *B*. *ephippium* (four males and two females), four *B*. *pitanga* (three males and one female) and three *I*. *parva* (all males). Individuals were lightly sedated with a topical application of benzocaine gel (10% or 20% depending on the animal size; Orajel, Church and Dwight Inc.) and sedation lasted during the entire course of the experiment, ensuring no electrical interference from muscle. One silver electrode (50 µm thickness) was inserted subcutaneously near the otic region in the earless *Brachycephalus* species and behind the tympanic membrane in *I*. *parva*. The second electrode was introduced into the nostril of the animal, on the same side as the otic electrode. Evoked potential responses measured with this method were as strong or stronger than responses measured with the second electrode inserted subcutaneously in the middle of the head. Nostril insertion was preferred for *Brachycephalus* species to reduce invasiveness of the procedure, due to their minute size and the lack of loose skin on top of their skull, which rendered electrode insertion difficult. The reference electrode was inserted either subcutaneously in the leg, or in the cloaca. Trials were performed in an acoustically isolated chamber. Sound was emitted by a Creative Loudspeaker D-100 placed 80 cm from the frog and calibrated by a G.R.A.S. ½ inch microphone placed 1 cm above the head of the frog. The microphone was connected to a G.R.A.S. 26AK preamplifier and G.R.A.S. 12AA power supply (G.R.A.S. Sound and Vibration, Holte, Denmark). Calibration of the microphone was performed re 94 dB at 1 kHz with a B&K Type 4231 calibrator (Brüel & Kjaer, Nærum, Denmark). Calibration, sound stimulation and ABR recording were controlled by custom-made software (QuickABR_burst) on a PC and digital sound processor (RM2, Tucker-Davis Technology, Alachua, FL).

Each trial started and ended with a series of click stimuli at 70–110 dB SPL, to ensure that the electrodes were correctly placed and that the responses had not deteriorated during the experiment. Clicks contain a broad range of frequencies and elicit ABR regardless of the sensitivity range of the ear. A series of pure tone bursts of duration 25 ms and ranging from 200 Hz to 6 kHz were broadcast at sound levels from below threshold to 110 dB SPL in 10 dB SPL steps. Evoked potential peak size increased with stimulus amplitude, generating a sigmoid response function at each stimulus frequency. From the response function the hearing threshold at this frequency was determined as the level at the intercept between a linear regression on the steep part of the curve and the noise level of the recording^18^. To produce hearing sensitivity threshold curves, generalized additive models (GAM) were fitted to the hearing sensitivity data with the R package *mgcv* ^34^.

### Laser Doppler Vibrometry (LDV)

Vibrational responses to acoustic stimuli were recorded in live specimens, including ten *B. ephippium*, 11 *B. pitanga* and nine *I. parva*. In preparation for the LDV experiments, specimens were cooled down in an ice-filled container to reduce activity. Frogs were placed on a platform of moistened cotton at a distance of 30 cm from the laser unit in as natural a posture as possible (Fig. 3 B), except for the ventral scans for which they were on their backs. Scans of lateral, dorsal and ventral surfaces of each frog were taken. Lateral surfaces of the animals were positioned at 90° perpendicular to the path of the laser. For scans of dorsal and ventral surfaces, a mirror was positioned above the specimen in the path of the laser beam, angled downwards at 45°. Between scans the specimens were mist-sprayed with water to maintain natural body conditions.

Vibrations of the surface of the skin in response to acoustic stimuli were recorded using a micro-scanning laser Doppler vibrometer (Polytec PSV-500; Waldbronn, Germany) fitted with a close-up attachment and 300 mm lens. This type of vibrometer does not require the use of reflective medium (beads or tape) and is thus non-invasive. A loudspeaker was positioned above the laser unit, facing the specimen. Sound stimuli were generated with the laser unit software (Polytec PSV 9.2) and passed to an amplifier (Sony Amplifier Model TAFE570; Tokyo, Japan), and subsequently to the speaker (neoX 2.0 True Ribbon Tweeter, Fountek Electronics Ltd, China). The stimuli were periodic chirps spanning frequencies from 1.5–20 kHz including the dominant frequency ranges of all species being measured. The stimuli were monitored and recorded as a reference at the position of the animal with a ¼ inch condenser microphone (G.R.A.S. 40BE, which has a frequency response of 30 Hz to 100 kHz according to the manufacturer). This allowed us to be certain of a frequency-flat stimulus of appropriate amplitude at the position of the animal. This microphone was connected to a G.R.A.S. 26AC preamplifier, and G.R.A.S. 26AA amplifier (G.R.A.S. Sound and Vibration, Holte, Denmark). Computer-controlled spectral correction of the acoustic stimulus was used to maintain a constant sound pressure level (±1.5 dB maximum deviation) across the complete range of frequencies. Acoustic stimuli were presented at 50 dB SPL at the position of the animal. Calibration of the reference microphone was performed re 94 dB at 1 kHz with a B&K Type 4231 calibrator (Brüel & Kjaer, Nærum, Denmark).

The LDV was used in scan mode. In the LDV software, a grid of scan points was defined on the surface of the positioned specimen. For the lateral scans, the area considered incorporated the entirety of the visible surface of the body, taking care to avoid directing the laser into the eyes. Efforts were made to include the otic region, the lung area and the mouth, as these areas have been previously implicated in sound transmission in certain frogs. Scans were also performed covering the legs and feet. For the dorsal scans, the entirety of the back covering at least the head and the lungs was defined. A density of at least 400 scan points was used per scan, depending on the size of the specimen and area to be scanned. The laser spot was ca. 5 µm in diameter, and the beam was spatially positioned with an accuracy of ca. 1 µm. In the frequency domain setting of the LDV, a frequency spectrum was calculated for every point using a FFT with a rectangular window, at a sampling rate of 128 kHz, 128 ms sampling time, and with a frequency resolution of 7.8125 Hz. A 500 Hz high-pass filter was applied to both the laser signal and the reference microphone during the scanning process.

To investigate low frequency vibration response, further experiments were carried out on four *B. ephippium*, one *B. pitanga* and one. *I. parva*. For these, a low-frequency-capable speaker (Audioengine2 speakers, Audioengine, Texas, USA) was used to broadcast periodic chirps spanning a range of 150 Hz to 10 kHz. Before working with the specimens, the reference microphone was placed at the position of the specimen (i.e., the same distance and angle of the frog to the speaker and laser). The stimulus was then flattened as before (±1.5 dB across all frequencies) and the amplitude adjusted to be at 50 dB. The flattened signal was then recorded for reference to calculate the transfer function of the frog response. The microphone was then removed and the specimen was placed at this position. Definition of scan points on the specimens was the same as in the previous experiments. The same FFT with rectangular window was analysed for every point, at a sampling rate of 25.6 kHz, 640 ms sampling time, and with a frequency resolution of 1.5625 Hz. A 150 Hz high-pass filter was applied to the returning laser signal during the scanning process. To control for vibrations of the experimental set-up, a single scan point of the platform in close proximity to where the specimen was placed was taken for post-recording correction of the laser signal.

Areas of high vibration amplitude, as well as any resonant tunings or specific frequency responses, were identified by analysis of vibration velocity and/or displacement of each scan point. Data from the LDV experiments were analysed using Polytec software (v 9.2). For the initial scans with the reference microphone at the position of the specimen, frequency spectra of the vibrometry data were normalized to those of the reference signal by computing the transfer function between the two. To observe a spatially continuous pattern of vibration, the single scan points in the grid were interpolated to produce vibration maps of the entire scanned area. This was done for graphical reconstructions of the data only and not for analysis of the single scan points. All experiments were performed in a sound-attenuating chamber (L2.4 × W1.8 × H2 m) on an anti-vibration table (L1 × W1 m, Melles Griot, Rochester, NY, USA) at temperatures of 22–25 °C.

### Micro-computed tomography

Middle ear morphology of *B. ephippium*, *B. pitanga* and *I. parva* was examined by combining micro-Computed Tomographic (µCT) scanning with diffusible iodine-based contrast-enhanced (DICE) staining of soft tissues. DICE staining method allows for better discrimination of non-ossified tissues such as muscle, cartilage or nerves in µCT scans^35^. Preserved specimens of *B. ephippium*, *B. pitanga* and *I. parva* were stained in 25% Lugol iodine solution for 24 hours and wrapped in cellophane to minimize drying during the scans, which were made using a Nikon XT H 225 µCT scanner. The settings used were 130 kV and 110 µA. Images were constructed from 1080 projections, each with 1000 ms exposure and two frames averaged per projection. The scan data were processed with CT AGENT XT 3.1.9 and CT PRO 3D XT 3.1.9 (Nikon Metrology, 2004–2013). Cubic voxel side-lengths were 14.5 μm. Tiff stacks from the micro-CT scans were converted to jpegs in Adobe Photoshop CS 8.0 (Adobe Systems Inc., 2003). Three-dimensional models of middle ears and surrounding structures (Fig. 1) were then reconstructed using WinSurf 4.0 (E. Neufeld, 2001).

### Histology

Specimens were euthanized with 20% benzocaine gel and fixed in 4% formaldehyde in PBS buffer or Karnovsky solution for 48 hours. They were subsequently transferred to 70% ethanol. After two to eight weeks of decalcification in a constantly spinning bath of 4% ethylenediaminetetraacetic acid (EDTA), specimens were gradually dehydrated using up to 100% ethanol and embedded in glycol methacrylate (Leica Historesin). Serial sections (4 µm thick) were made with a microtome (Leica RM2255) using glass knifes. Histological sections were stained with toluidine blue and fuchsine to reveal cellular structures. Photomicrographs were obtained using an Olympus BX51 microscope equipped with a digital camera and Image-Pro Express software, version 5.0 (Media Cybernetics). These photomicrograph stacks were automatically aligned via rigid-body translation and rotation using ImageJ^36^ 1.45 s, running the Stackreg plugin^37^. Any obvious alignment errors were manually corrected using Adobe Photoshop CS 8.0 (Adobe Systems, 2003). Three-dimensional models of middle and inner ear structures were then reconstructed using WinSurf 4.0 (E. Neufeld, 2001). Schematic representations of inner ear structures of interest (Fig. 4a,b and c) were made by M.J.M. based on these three-dimensional models.

## Electronic supplementary material

Supplementary information

Video S1. Male Brachycephalus pitangaBrachycephalus pitanga vocalizing in its natural habitat

Video S2. Arm-waving and mouth-gaping behaviours in female Brachycephalus pitangaBrachycephalus pitanga
