# Supplementary material for: Evidence of auditory insensitivity to vocalization frequencies in two frogs
Source: Sci Rep. 2017 Sep 21;7:12121. doi: 10.1038/s41598-017-12145-5 (PMC5608807; doi:10.1038/s41598-017-12145-5)
Supplement: Supplementary file 1 — Supplementary information [file 41598_2017_12145_MOESM1_ESM.pdf]

Supplementary information for *Evidence of auditory insensitivity to vocalisation frequencies in two frogs*

Sandra Goutte[a,*], Matthew J. Mason[b], Jakob Christensen-Dalsgaard[c], Fernando Montealegre-Z[d], Benedict D. Chivers[d], Fabio A. Sarria-S[d], Marta M. Antoniazzi[e], Carlos Jared[e], Luciana Almeida Sato[e], Luís Felipe Toledo[a]

[a] Laboratório de História Natural de Anfíbios Brasileiros (LaHNAB), Departamento de Biologia Animal, Instituto de Biologia, Universidade Estadual de Campinas, Campinas, São Paulo, 13083-862, Brazil.

[b] Department of Physiology, Development & Neuroscience, University of Cambridge, Downing Street, Cambridge CB2 3EG, United Kingdom.

[c] Department of Biology, University of Southern Denmark, Campusvej 55, DK-5230 Odense M, Denmark.

[d] Bioacoustics and Sensory Biology Lab, Joseph Banks Laboratories, University of Lincoln, Green Lane, Lincoln, LN6 7DL, United Kingdom.

[e] Laboratory of Cell Biology, Instituto Butantan, São Paulo, 05503-900, Brazil.

*corresponding author: s.m.goutte@gmail.com, +33 6 79 20 29 99.

Supplementary figures

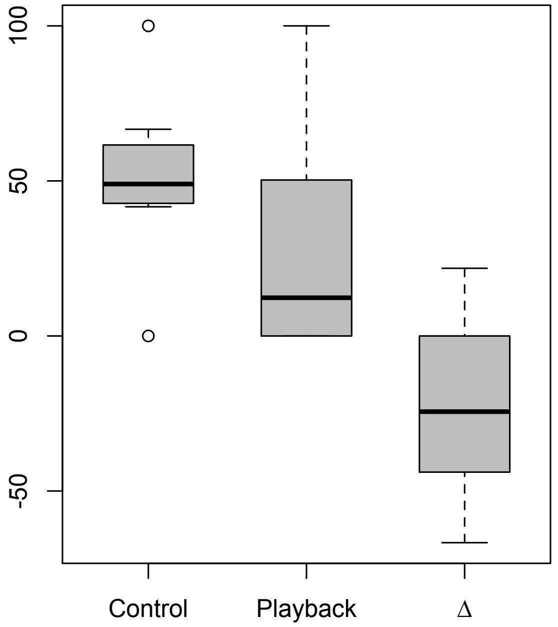

Figure S1. Calling activity in male *Brachycephalus pitanga* during playback. Percentage of time spent calling during control and playback in male *Brachycephalus pitanga* (n=8). The difference in calling activity between playback and control within males is given (Δ). The difference in calling activity between the two treatments is not significant (Paired t-test t=2.20, p-value = 0.06), although a there is a tendency: calling activity is reduced during playback for most males (negative Δ values), as males were selected because they were spontaneously calling and the control treatment was done first. No behaviour such as orientation towards the speaker or phonotaxis was observed during playback.

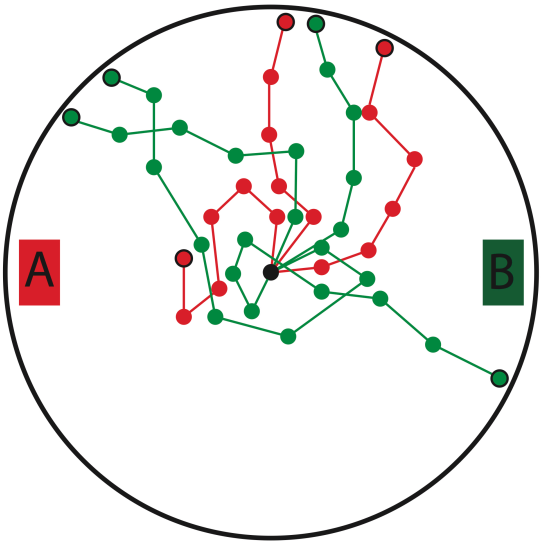

Figure S2. Phonotaxis experiment in female *Brachycephalus pitanga*. Trajectories employed by the female *Brachycephalus pitanga* (n=7) within the circular arena during playback. Rectangles represent speakers A (red) and B (green) from which specific calls were broadcast. Each trajectory is represented by full circles joined by straight lines and colour-coded red or green according to the speaker playing during the trial. Coloured circle with a black outline represent the position of the frog at the end of the trial (when the female exited the arena or if she had stopped for more than ten minutes).

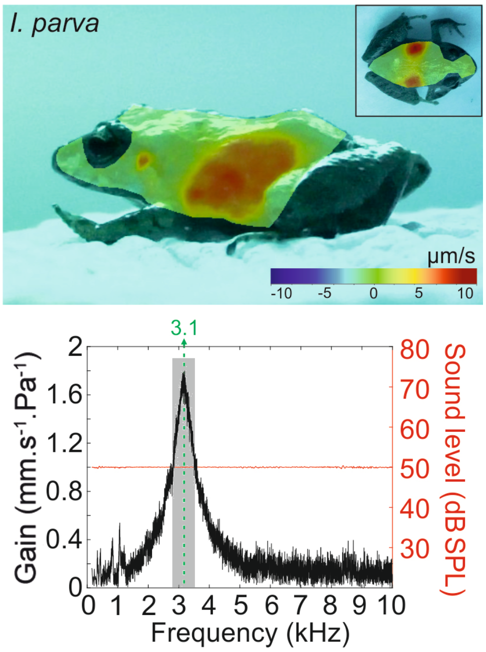

Figure S3. Deflection pattern of skin vibrational response to airborne sounds (1.5-20 kHz) of *Ischnocnema parva*. Areas of high vibration amplitude (tympanic membrane and skin overlying the lungs) are indicated in red colours; a velocity colour scale is given (upper panel). Skin vibrational response presented as velocity gain (transfer function of the laser signal and stimulus reference) from 150 Hz to 10 kHz (lower panel). The advertisement call frequency range (2.87-3.53 kHz) is shaded in grey.

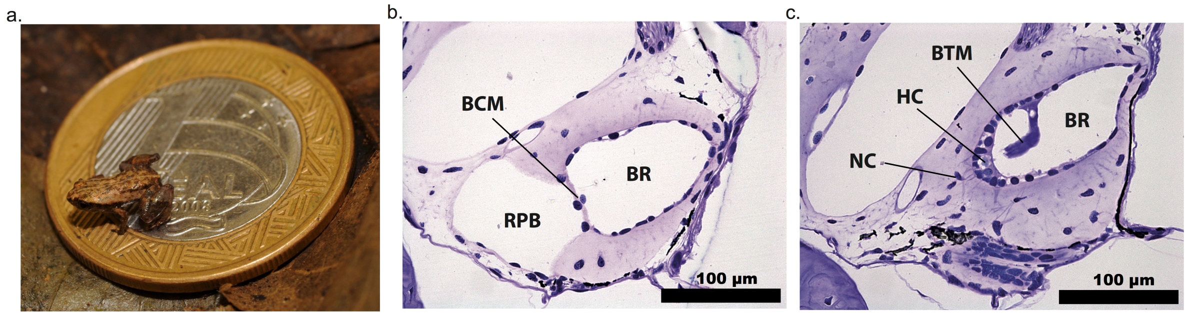

Figure S4. Morphology and inner ear structures in *Brachycephalus hermogenesi*. a. Adult *B. hermogenesi* in life on a Brazilian one real coin (diameter 2.6 cm), b. and c. Photomicrographs of *B. hermogenesi* inner ear structures. BCM: basilar recess contact membrane, BR: basilar recess, BTM: basilar recess tectorial membrane, HC: hair cells, NC: eighth cranial nerve cells, RPB: *recessus partis basilaris*.

Supplementary videos

Video S1. Male *Brachycephalus pitanga* vocalizing in its natural habitat. Although vocalizations (very quiet rasping sounds) are hardly distinguishable from the background noise and are masked by bird songs, the inflations and deflations of the vocal sac are clearly visible.

Video S2. Arm-waving and mouth-gaping behaviours in female *Brachycephalus pitanga*. Sequence of 20 photos (4 ms duration per frame) taken in the laboratory. In the background, two eggs laid by the same individual on the previous night.
